# Dynamic environments do not appear to constrain spider web building behaviour

**DOI:** 10.1007/s00114-021-01725-1

**Published:** 2021-04-29

**Authors:** Tom Mulder, Lucas Wilkins, Beth Mortimer, Fritz Vollrath

**Affiliations:** Department of Zoology, University of Oxford, 11 Mansfield Road, Oxford, OX1 3SZ UK

**Keywords:** Garden cross spider, *Araneus diadematus*, Silk tension, Web distortion, Web building, Web geometry

## Abstract

Many laboratory experiments demonstrate how orb-web spiders change the architecture of their webs in response to prey, surroundings and wind loading. The overall shape of the web and a range of other web parameters are determined by frame and anchor threads. In the wild, unlike the lab, the anchor threads are attached to branches and leaves that are not stationary but move, which affects the thread tension field. Here we experimentally test the effect of a moving support structure on the construction behaviour and web-parameters of the garden cross spider *Araneus diadematus.* We found no significant differences in building behaviour between rigid and moving anchors in total time spent and total distance covered nor in the percentage of the total time spent and distance covered to build the three major web components: radials, auxiliary and capture spirals. Moreover, measured key parameters of web-geometry were equally unaffected. These results call for re-evaluation of common understanding of spider webs as thread tensions are often considered to be a major factor guiding the spider during construction and web-operation.

**Supplementary Information:**

The online version contains supplementary material available at 10.1007/s00114-021-01725-1.

## Introduction

**Fig. 1:**
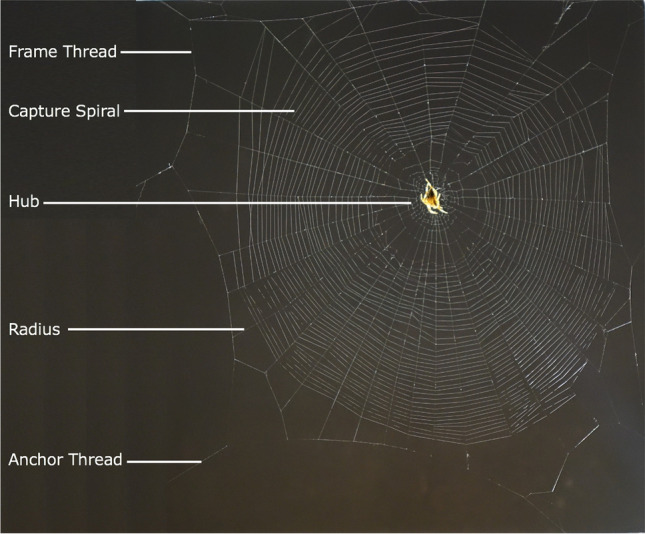
Main features of an *Araneus diadematus* orb web

Numerous studies have examined the intricate features of spider orb webs typically concluding that the features of orb webs are largely dictated by their function as prey traps (Fig. 1) (Burtscheidt et al. 2019; Dyson 2018; Krink and Vollrath 2000; Mortimer et al. 2014; Nyffeler 2009; Pasquet et al. 2013; Rhisiart and Vollrath 1994; Schneider and Vollrath 1998; Souza et al. 2007; Uetz et al. 1978; Vollrath et al. 1997; Wu et al. 2013; Zschokke 2011). For example, in vertical webs, the spiders build larger capture areas below the hub because they can run faster down than up, which encourages them to expand that area of their effective control (Rhisiart and Vollrath 1994; Watanabe 2000; Zschokke 2011). Whilst this functionality is easily tested, much more difficult to study are the effects of internal and environmental factors and the constraints they might impose on webs (Anotaux et al. 2016; Dyson 2018; Hesselberg 2013; Mazzia et al. 2020; Pasquet et al. 2013; Schneider and Vollrath 1998; Tew and Hesselberg 2018, 2017; Vollrath et al. 1997; Wolff et al. 2020; Wu et al. 2013). For example, in windy conditions, spiders like the garden cross spider *Araneus diadematus* alter many web features (presumably in order to minimise wind damage) such as total web area, capture spiral area, web eccentricity, mesh space, capture spiral count, radial count, total radial length and total capture spiral length (Vollrath et al. 1997; Wu et al. 2013; Fig. 1). In another example, in constrained spaces, *A. diadematus* modifies many inter-connecting key features of web architecture again showing the high degree of flexibility in the spider’s web-construction algorithm (Krink and Vollrath 2000).

Whilst web building in spatially constrained and windy and environments demonstrates the spider’s ability to adjust to  rather static conditions, in nature the supporting structures often move relative to each other due branches swaying in the wind (Online Resource 1, Online Resource 2). Despite the common occurrence of this environmental disturbance, the effects of moving anchors on orb web building behaviour and orb web design stand unexamined. The potential effects of moving anchors are particularly interesting because spiders carefully manage silk tensions in their webs during construction (Eberhard 1981; Mortimer 2019; Watanabe 2000; Wirth and Barth 1992; Zschokke 1996) and independent anchor movements should cause continuous tensions changes in any established threads. Studying the effects of moving anchors may additionally demonstrate whether spiders dedicate more time consolidating structural components of webs in mechanically taxing (non-windy) environments, and if the strong association between wind and anchor movement in nature causes spiders to change their webs as they do in windy conditions.

Given these unknowns, the present study aimed to explore whether orb weavers alter their building behaviour and hence modify resulting web structures in the laboratory when anchors move continuously before, after and throughout the building period. We hypothesised that, when faced with moving anchors: (i) web construction would take longer, (ii) a greater percentage of the total construction time would be dedicated to structural components (radials, auxiliary spiral), (iii) the construction path would be more tortuous and (iv) web geometry might be adjusted comparable to builds in windy conditions.

## Materials and methods

### Spider preparation

Adult female *Araneus diadematus* were collected from several locations in Oxfordshire, England. Spiders were stored in our standard Perspex frames (30 cm × 30 cm × 5 cm) separated by greased Perspex sheets. Twice per week, they were watered with a spray gun and fed two *Drosophila melanogaster*. Spiders had to build three trial webs on consecutive days in standard frames to be able to be selected for experiments.

### Experimental method

**Fig. 2:**
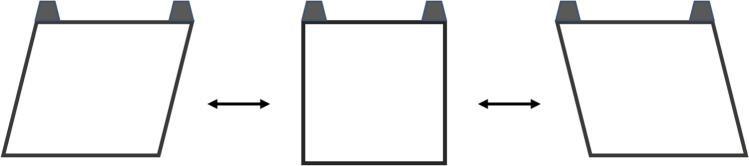
Shaker induced movement of flexible frame. Dark blocks fixed flexible frames in shaker. When a frame was attached to a mechanical arm, the bottom panel of the frame shifted ~ 10 cm left and right at ~ 0.05 Hz whilst the top panel remained fixed in place. Higher moving frequencies were attempted but caused spiders to stop building webs altogether. All attached flexible frames moved in unison due to the parallel configuration. To control for motor-induced vibrations in the shaker, the front frame in the shaker was always empty and moving

Spiders were transferred into individual flexible frames which are identical to standard frames but with bendable corners. Four flexible frames were placed in parallel in the frame-shaking tool (henceforth ‘shaker’). An electric motor moved 4 parallel mechanical arms, each of which could be attached to the bottom of a flexible frame with magnets. Under Rigid control conditions ‘*R*’, the flexible frame was detached from the moving mechanical arm and remained stationary. Under Moving treatment conditions ‘*M*’, the frame was attached to its arm and was repeatedly moving side to side (Fig. 2). To establish a workable frequency and configure the hardware, we cycled through circa 10 spiders (in addition to those used in the actual experiment) in a set of pilot trials, some of which were used for multiple trial settings. Ultimately, it was not possible to fully and consistently replicate the variability of anchor movements in nature (Online Resource 1), and during trials spiders refused to build webs altogether at frequencies > 0.05 Hz. The results have been interpreted cautiously to reflect these experimental constraints.

The experiments followed a 5-day *RRMRM* regime. This *RRMRM* regime is known as (multiple baseline) reversal design and is particularly suitable for small N research designs (Saudargas and Drummer 1996). We selected this design because we predicted that our challenging experiment would result in a small sample size. Put succinctly, the reversal design ensures that observed effects are unlikely to be caused by extraneous factors as it increases internal validity (Saudargas and Drummer 1996)and thus (partly) compensates for a small sample. On days where spiders did not build a web, the same run was repeated up to four consecutive days (*e.g. R*^*1*^*R*^*2*^*R*^*2*^*R*^*2*^*R*^*2*^*M*^*3*^*R*^*4*^*M*^*5*^) after which the spider was removed and the sequence was considered incomplete. Between runs, the webs were sprinkled with water and all radials were cut except for two radials leading North and South to the frame. Spiders were fed 1 *D. melanogaster* per day of the experimental regime regardless of web building success and experimental conditions.

#### Building behaviour

Web building was recorded with timelapses; one time-stamped photograph was obtained per ~ 10 s. With a custom tracking programme in Python, the coordinates of spiders and all frame corners were recorded for each photograph. Tracking began when spiders started web construction and terminated when the spider sat still at the hub. Raw tracks were corrected for shaker-induced movements in moving runs—because frame movements were greatest at the bottom, the correction was adjusted for the spider’s latitudinal position in the frame. By scaling relative to the frames, the absolute distances between corrected coordinates were calculated.

From activity plots (Online Resource 3) and established activity signatures from (Zschokke and Vollrath 1995), we identified the photographs in which spiders started and completed construction of the radials, auxiliary spiral and capture spiral. This information was used to calculate four building behaviour variables; time differences between photographs were used to calculate the total web construction time and the percentage of time spent on each web component, and the absolute positional data were used to calculate the total distances covered during total web construction and the percentage of the distance covered per web component.

#### Web geometry

The resulting webs were photographed (Panasonic LUMIX GH5 digital camera and Nikon AF NIKKOR 50 mm lens) and eight web measurements were obtained in ImageJ (Online Resource 4); measurements scaled relative to frames. These measurements were used to calculate the six wind-affected web features (Table 1). Whilst the total radial lengths and capture spiral length are also affected by wind (Vollrath et al. 1997), we did not include these features in the analysis as they are geometrically related to other included features; radial length is determined by web area and radial count, whilst capture spiral length is determined by capture spiral count, mesh space and capture spiral area.

**Table 1:** Analysed web design features

Features	Calculation (if applicable)
Total web area (cm^2^)	N/A (measured directly)
Capture spiral area (cm^2^)	$=\mathrm{Hub and capture spiral area}-\mathrm{hub area}$
Eccentricity*	${=\sqrt{\left(1-\frac{\mathrm{Web width}}{\mathrm{ Web height}}\right)}}^{2}$
Mean capture spiral count**	$=\frac{\left(\mathrm{North capture spiral count }+\mathrm{ South capture spiral count}\right)}{2}$
Mean mesh space** (cm)	$=\frac{\left(\mathrm{Width north capture spiral }+\mathrm{ Width south capture spiral}\right)}{(\mathrm{Count north capture spirals}+\mathrm{ Count south capture spirals}-2)}$
Radial count	N/A (measured directly)

*Features calculated per the methods in Vollrath et al. (1997), **features calculated per the methods in Wu et al. (2013). See Online Resource 4 for example photographs of how the measurements were taken from webs. North, East, South and West indicate directions relative to the hub. We calculate web width = length east radius + length west radius, and web height = length north radius + length south radius

### Data analysis

The effects of moving anchors on web design features and building behaviour were examined with mixed models (Davies and Gray 2015) in which Spider ID was included as a random effect to adjust for pseudoreplication. The models thus examined whether anchor movement affected building behaviour and each web feature when adjusted for variation that naturally occurs between individuals.

Crucially, spiders may also use the experience of building one web under certain conditions when building the following web *e.g.* (Venner et al. 2000). Using boxplots, we visually assessed if there was an experience effect within the rigid data (days R1, R2, R3) or within the moving data (days M1, M2) for any of the variables of interest. The boxplots did not indicate a clear and significant experience effect (Online Resource 5–7).

The effects of moving anchors on capture spiral count and radial count data were assessed with generalised linear mixed models (GLMMs); Poisson error distributions. All other dependent variables were continuous and analysed with linear mixed models (LMMs). Several continuous variables analysed with LMMs were log-transformed or inverse transformed to meet the assumption of normality which was verified with histograms, Q-Q plots and Shapiro Wilk tests (Online Resource 8).

Multiple LMMs and the radial count GLMM were overfitted (*i.e.* not optimally parsimonious (Hawkins 2004)) due to the inclusion of the random effect. However, because overfitting did not change model outputs at all, we opted to retain Spider ID in all models to accurately represent our experimental design throughout. Per the methods in (Thomas 2017), the capture spiral count and radial count GLMMs were also assessed for overdispersion (when variability in the data exceeds that predicted by the Poisson GLMM (Berk and MacDonald 2008)), and no significant overdispersion was detected (ratio = 0.69, *p* = 0.82 and ratio = 0.34, *p* = 0.99 respectively). The assumptions of all models were met.

Finally, as we examined four building behaviour variables, a Bonferroni correction was applied and $p \le 0.013$ would indicate a significant effect. Likewise, because we examined six web design features, $p \le 0.008$ would indicate a significant effect.

## Results

### Sample size

At a frame movement frequency of 0.05 Hz, the experiment was attempted with 26 spiders, of which only five spiders completed the full RRMRM experimental regime during the 11-month experimentation period. The small sample was thus not a consequence of spider availability, but instead, the experiment was limited by time and space availability in the shaker coupled with operational (filming) constraints.

The five successful spiders allowed us to obtain 25 timelapses (*n*_rigid_ = 15, *n*_moving_ = 10), but 5 webs were broken upon removal from the shaker due to static and were not photographed for web measurements (*n*_rigid_ = 11, *n*_moving_ = 9). On one occasion, a spider remained stationary for multiple extended periods partway through the build resulting in a total construction time of 15119 s, more than twice as long as the next longest construction time by any spider (6805 s). As this behaviour was abnormal and created an extreme outlier in terms of web construction time, this run was excluded from the temporal web building analyses. This run was not excluded from the web design analyses and spatial web building analyses because the resulting web design and distances covered were not affected by this behaviour.

### Web building behaviour

**Fig. 3:**
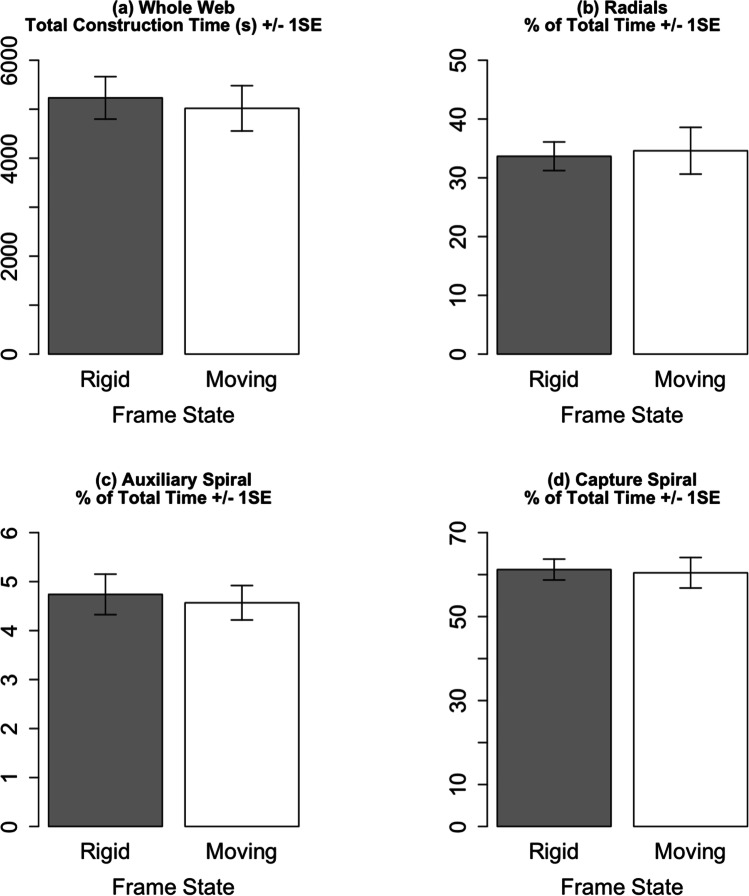
Effect of moving anchors on web construction times. SE = standard error. Effect sizes for models with log-transformed and inverse-transformed dependent variables were calculated by back transformation of model outputs. *n*_rigid_ = 15, *n*_moving_ = 9. See Online Resource 5 for relevant boxplots indicating a lack of an experience effect

Anchor movement did not significantly affect the construction times (df = 18.2, *t* =  − 0.46, *p* = 0.65) (Fig. 3a), or the percentage of the construction time spent on each web component (radials df = 22.0, *t* = 0.24, *p* = 0.82; auxiliary spiral df = 18.0, *t* = 0.43, *p* = 0.67; capture spiral df = 18.5, *t* =  − 0.21, *p* = 0.83) (Fig. 3b–d).

**Fig. 4:**
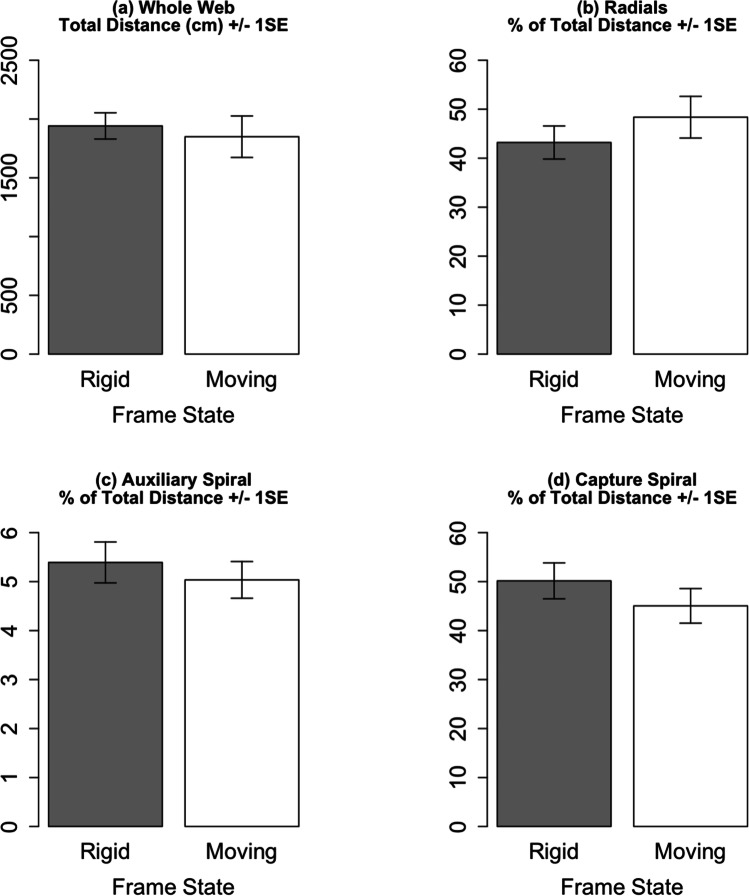
Effect of moving anchors on distances covered during web construction. SE = standard error. Effect sizes for models with log-transformed and inverse-transformed dependent variables were calculated by back transformation of model outputs. *n*_rigid_ = 15, *n*_moving_ = 10. See Online Resource 6 for relevant boxplots indicating a lack of an experience effect

Moving anchors also did not significantly affect the distances spiders covered (df = 23.0, *t* =  − 0.52, *p* = 0.61) (Fig. 4a), or the percentage of the total distance covered per web component (radials df = 19.0, *t* = 1.21,* p* = 0.24; auxiliary spiral df = 19.0, *t* = 0.82, *p* = 0.42) (Fig. 4b–d).

### Web geometry

**Fig. 5:**
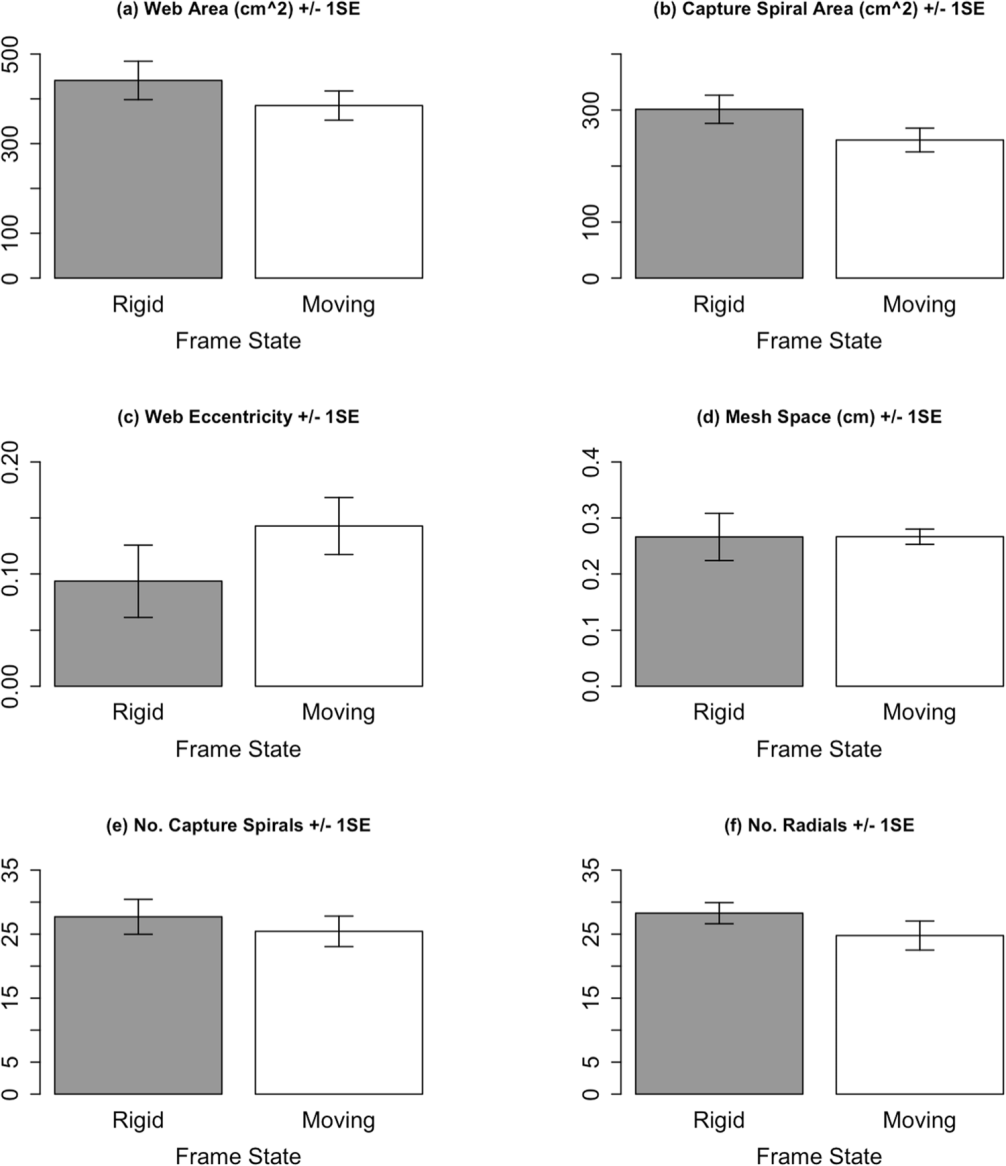
Effect moving anchors on web geometry. SE = standard error. True effect sizes for **d**–**f** were calculated by back transformation of model outputs. *n*_rigid_ = 11, *n*_moving_ = 9. See Online Resource 7 for relevant boxplots indicating a lack of an experience effect

The area of whole webs and area of capture spirals were not significantly affected by anchor movement (web area df = 14.4, *t* =  − 1.72, *p* = 0.11; capture spiral area df = 14.4, *t* = -2.58, *p* = 0.02) (Fig. 5a,b). Likewise, the anchor movements did not significantly affect web eccentricity and mesh space (eccentricity df = 14.4, *t* = 1.93, *p* = 0.07; mesh space df = 14.1, *t* = 0.03, *p* = 0.98) (Fig. 5c,d) or the number of capture spirals and radials in webs (capture spiral *z* =  − 0.96, *p* = 0.34, radials *z* =  − 1.50, *p* = 0.13) (Fig. 5e,f).

## Discussion

Our treatment considerably sheared the web during construction by sinusoidal moving of the anchor points by approximately 40° between the two maxima. Surprisingly, we found no significant effects of the shearing on both web building and a range of selected web features shown to be affected by blowing wind (Figs. 3, 4 and 5). This was unexpected because it is generally accepted that during web construction spiders regulate tensions (Eberhard 1981; Mortimer 2019; Watanabe 2000; Wirth and Barth 1992; Zschokke 1996) and our frame movements clearly affected the tensions in a cyclical fashion. Moreover, due to the common co-occurrence moving anchors and windy conditions in nature, one may have expected our artificial anchor movements to affect wind-sensitive web features, especially since our artificial anchor movements were slower than those observed in nature (Online Resource 1).

However, it must be noted that whilst the statistical tests did not detect a significant effect of anchor movement on web building behaviour and web features, the bar charts of several web features do indicate an influence of web movement (Fig. 5). A larger sample may have resulted in a significant effect of anchor movement on specific web features. Furthermore, because our analysis only included those individuals which completed the entire RRMRM regime, we likely selected the most robust web builders in our captive population. Our exclusive use of these robust builders may have resulted in smaller effect sizes and the observed non-significant effects.

Our experiments nonetheless suggest that repetitive changes in thread tensions during web-construction may be balanced-out by the spider. There is some evidence—from rotation and thread-cutting experiments—that orb spiders can average rapid modifications of sensory input (Reed 1969; Vollrath 1988). This mechanism allows the spider to use a robust ‘rule of thumb’ algorithm and avoid being confused by sensory input that might conflict with the expectations in the feed-back loop (Krink and Vollrath 2000). In a cybernetic control-theory analogy, this would amount to controlling a mismatch between measured values and set-points. Web-building is a dangerous process and rapid conclusion is of essence (Rypstra 1984; Vollrath 1992, 1980; Zschokke and Vollrath 1995). Final fine-tuning by adjusting the tensions once web-building is done is one way of finishing the trap (Mortimer et al. 2016; Watanabe 2000), although it appears that spiders can also locate prey using vibrations in webs that are distorted, and in which tensions are changed (Mulder et al. 2020).

We thus cautiously conclude that thread tensions are important but perhaps not as important as typically assumed at least whilst building the web. Notably, the visco-elastic properties of the silk will in itself contribute to the tension-landscape of a web both during and after building and it seems that our artificial anchor movements were not mechanically taxing on the structure of the web. However that might be, this study demonstrates, once again, that there is yet much more to study before we can say that we understand even the basics of spider webs and their construction process.

## Supplementary Information

Below is the link to the electronic supplementary material.

Online Resource 1 Video (180fps) demonstrating wind-induced movement of anchors in nature and consequent web deformations. Cornstarch was applied to the web for visibility purposes (MP4 122027 kb)

Online Resource 2 Web movement track data obtained from webs filmed at 180fps in nature. Tracked points include the centre of the hub, and one point in each web quadrants (North, East, South, West). Tracking was accomplished with a custom tracker programmed in Python (CSV 3762 kb)

Online Resource 3 Example activity plot indicating activity signatures as established by Zschokke and Vollrath (1995). Rigid conditions (PDF 384 kb)

Online Resource 4 Web-design measurements obtained in ImageJ. **a** total web area, **b** hub + capture spiral area, **c** hub area, **d** radial length North (yellow), East (green), South (red) West (blue), **e** capture spiral count North and South (demonstrating North 1–4), **f** capture spiral width North (yellow) and South (red), **g** radial count (demonstrating 1–9) (PDF 3367 kb)

Online Resource 5 Boxplots demonstrating the lack an experience effect on total web construction time and the percentage of time dedicated to each web component. Treatment = the specific day within the 5-day experimental regime (*R*^1^*R*^2^*M*^1^*R*^3^*M*^2^) in which the frame is either rigid (*R*) or moving (*M*). *R*-days and *M*-days are shown sequentially for easy comparison within each treatment group. Plots are based on raw, non-transformed data. The temporal outlier was excluded per the methods on page 11. *n*_rigid_ = 15, *n*_moving_ = 9 (PNG 147 kb)

Online Resource 6 Boxplots demonstrating the lack an experience effect on total distance covered during web construction, and the percentage of total distance covered for each web component. Treatment = the specific day within the 5-day experimental regime (R^1^R^2^M^1^R^3^M^2^) in which the frame is either rigid (R) or moving (M). R-days and M-days are shown sequentially for easy comparison within each treatment group. Plots are based on raw, non-transformed data. *n*_rigid_ = 15, *n*_moving_ = 10 (PNG 179 kb)

Online Resource 7 Boxplots demonstrating the lack an experience effect on six web geometry features. Treatment = the specific day within the 5-day experimental regime (*R*^1^*R*^2^*M*^1^*R*^3^*M*^2^) in which the frame is either rigid (*R*) or moving (*M*). *R*-days and *M*-days are shown sequentially for easy comparison within each treatment group. Plots are based on raw, non-transformed data. *n*_rigid_ = 11, *n*_moving_ = 9 (PNG 183 kb)

Online Resource 8 Data transformation employed per variable and Shapiro Wilk test results of LMM residuals. *Log* log-transformed, *Inv* inverse transformed (*i.e. x*^-1^). Where no transformation is indicated, data were not transformed (PNG 159 kb)

## Data Availability

Data available from the corresponding author upon reasonable request.

## Code availability

Statistical code and plots developed in RStudio. Code available from the corresponding author upon reasonable request.
