# Supplementary figures and images for: Dynamic environments do not appear to constrain spider web building behaviour
Source: Naturwissenschaften. 2021 Apr 29;108(3):20. doi: 10.1007/s00114-021-01725-1 (PMC8084787; doi:10.1007/s00114-021-01725-1)

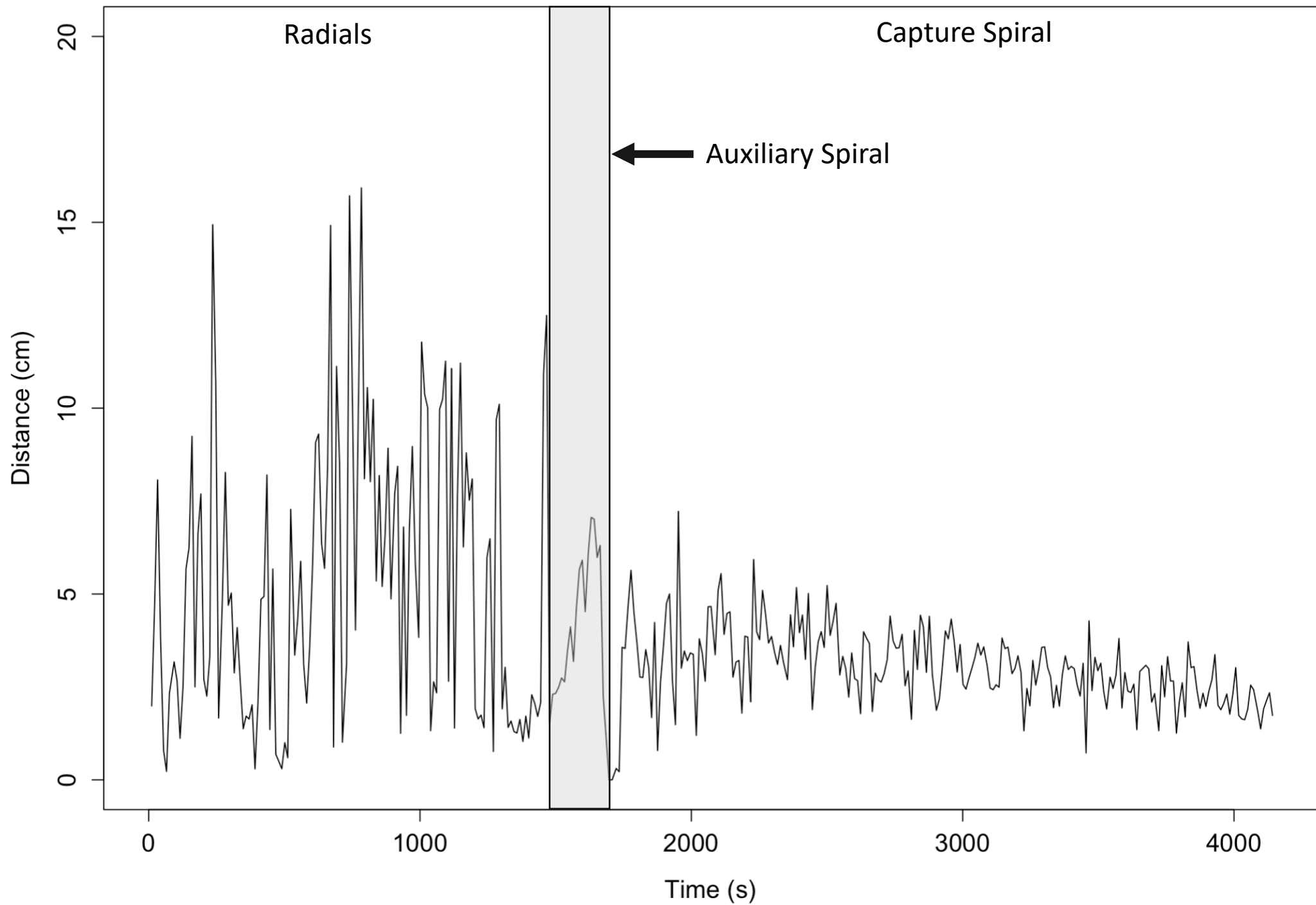

Supplement: Supplementary file 3 — Example activity plot indicating activity signatures as established by Zschokke and Vollrath (1995). Rigid conditions (PDF 384 kb) [file 114_2021_1725_MOESM3_ESM.pdf]

a

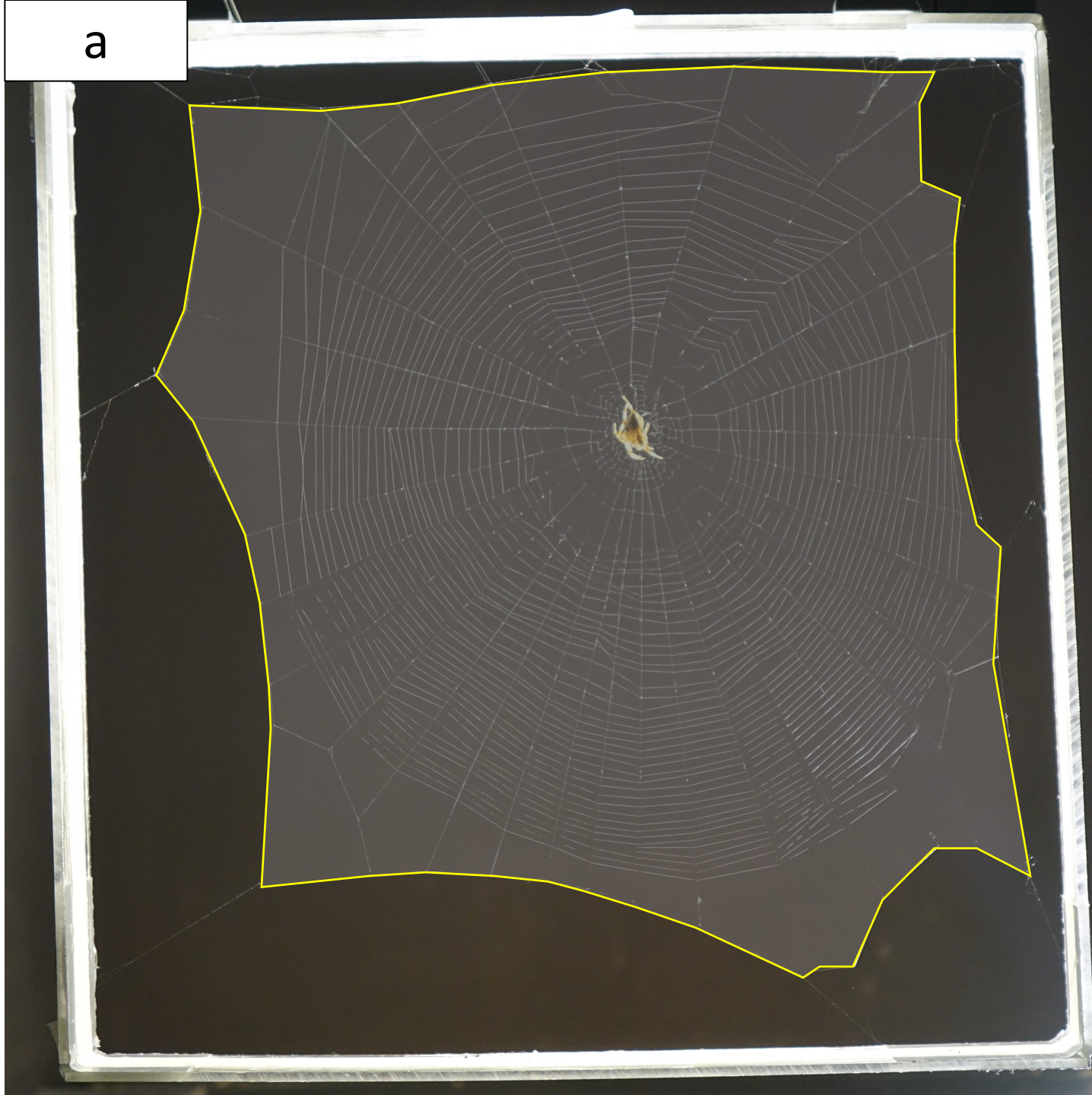

b

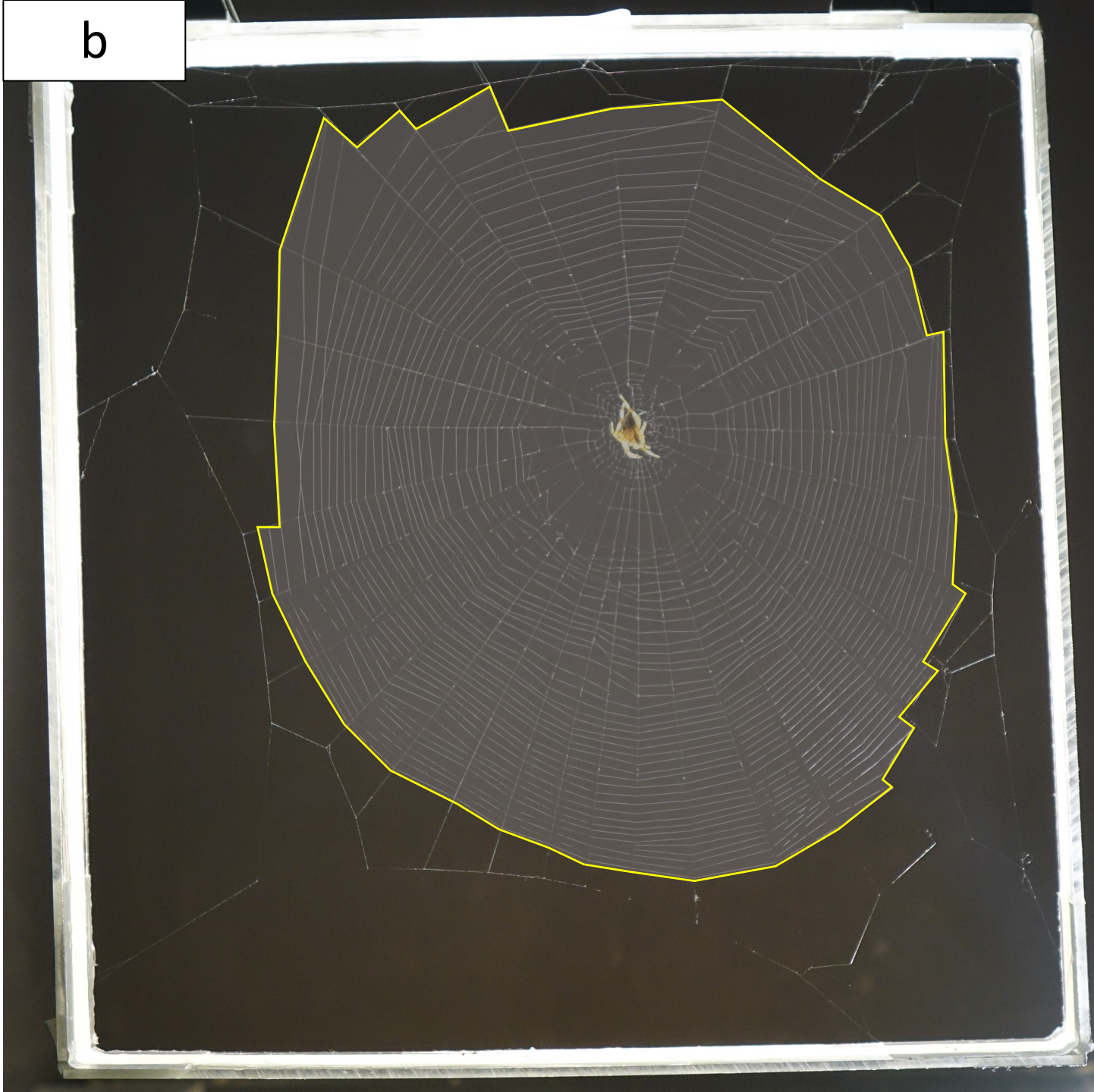

C

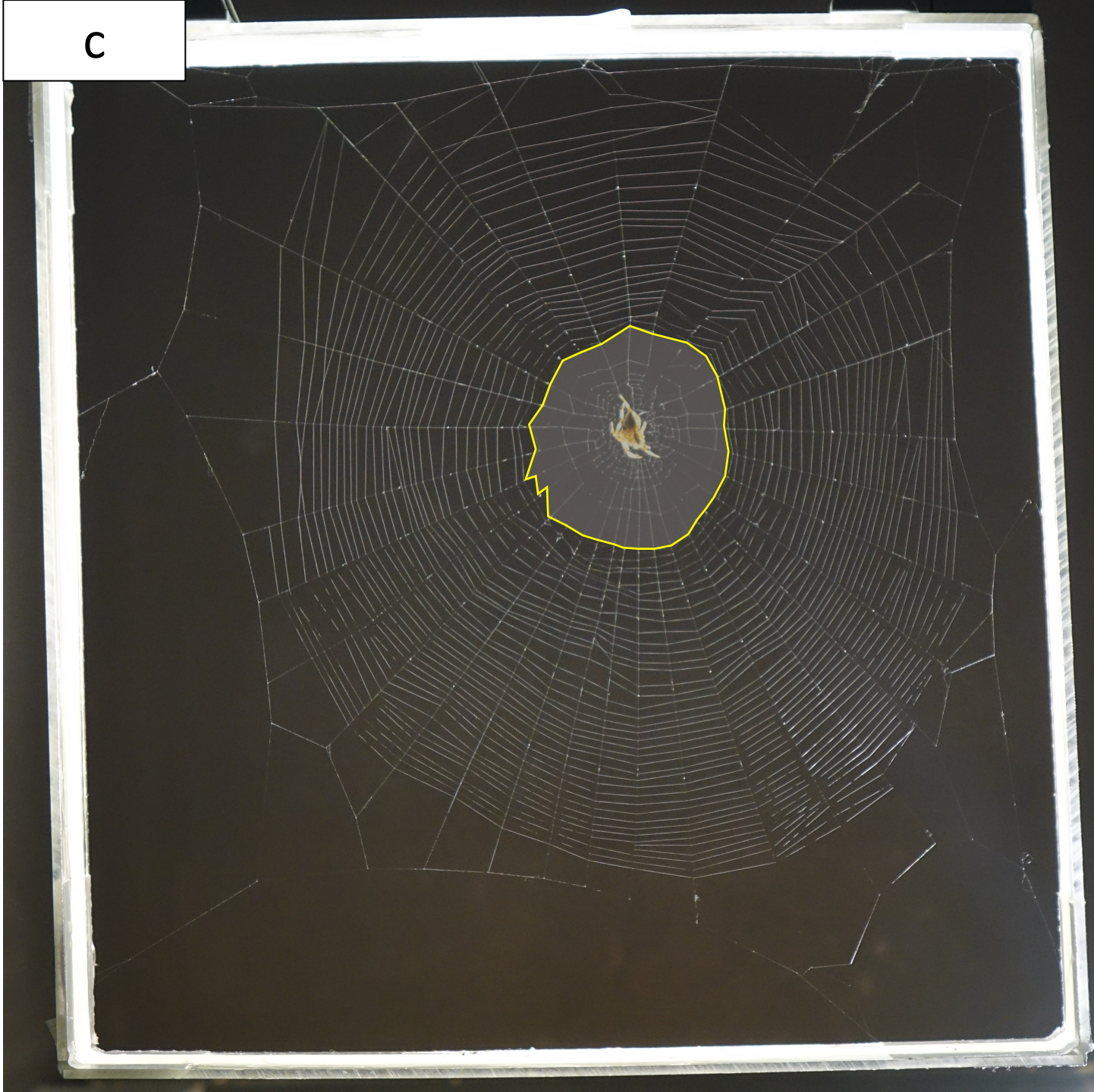

d

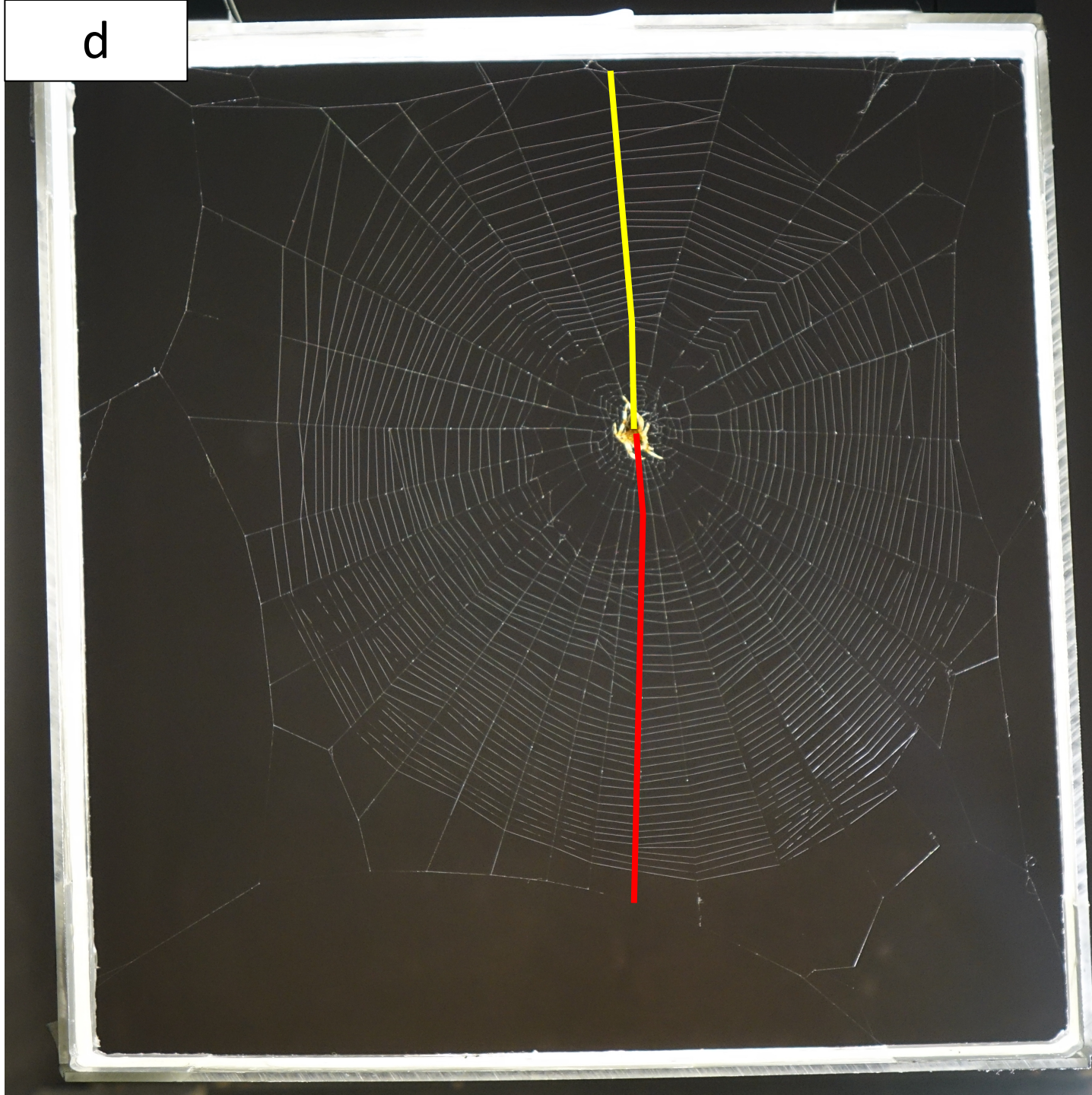

e

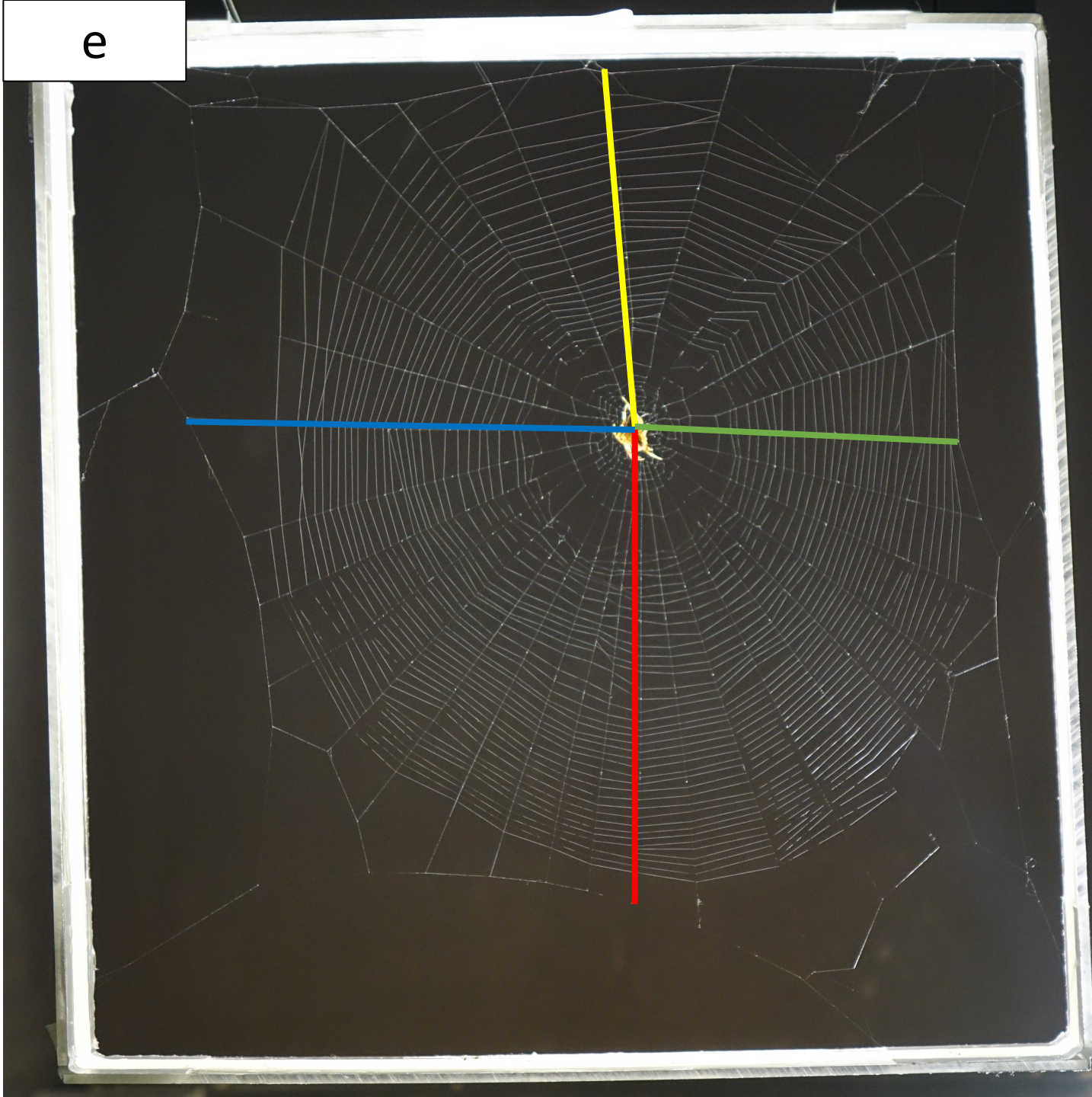

f

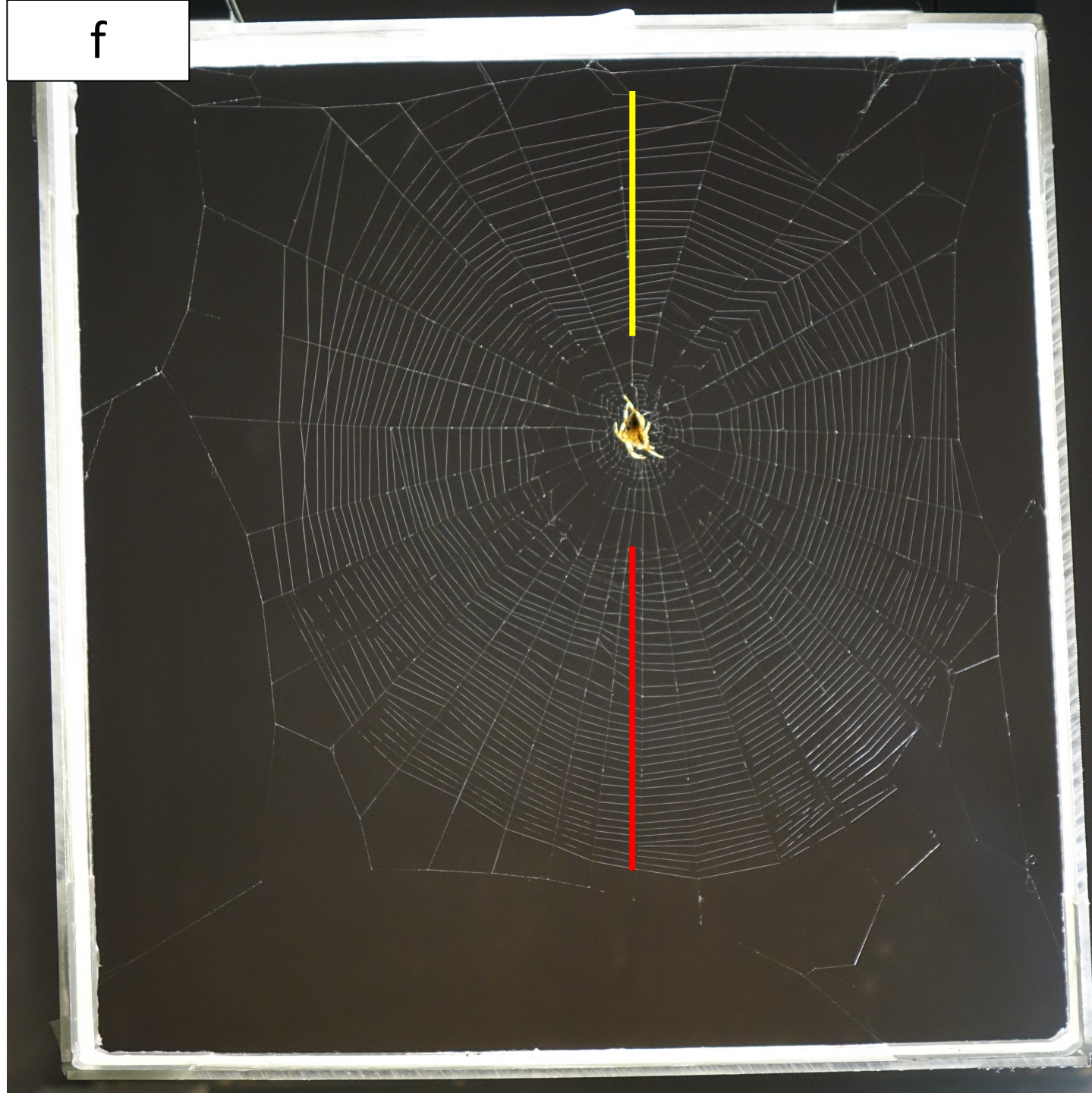

g

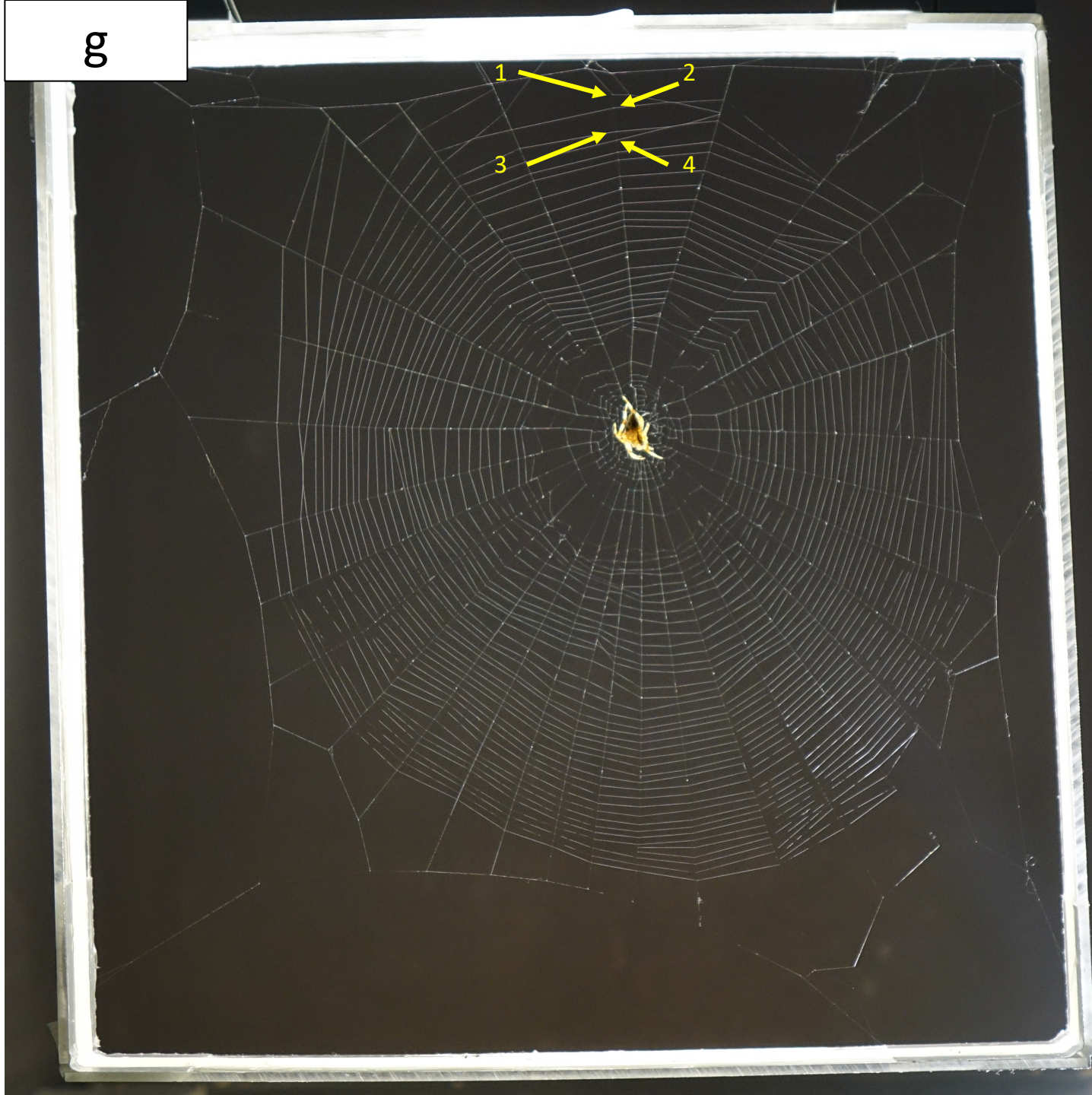

h

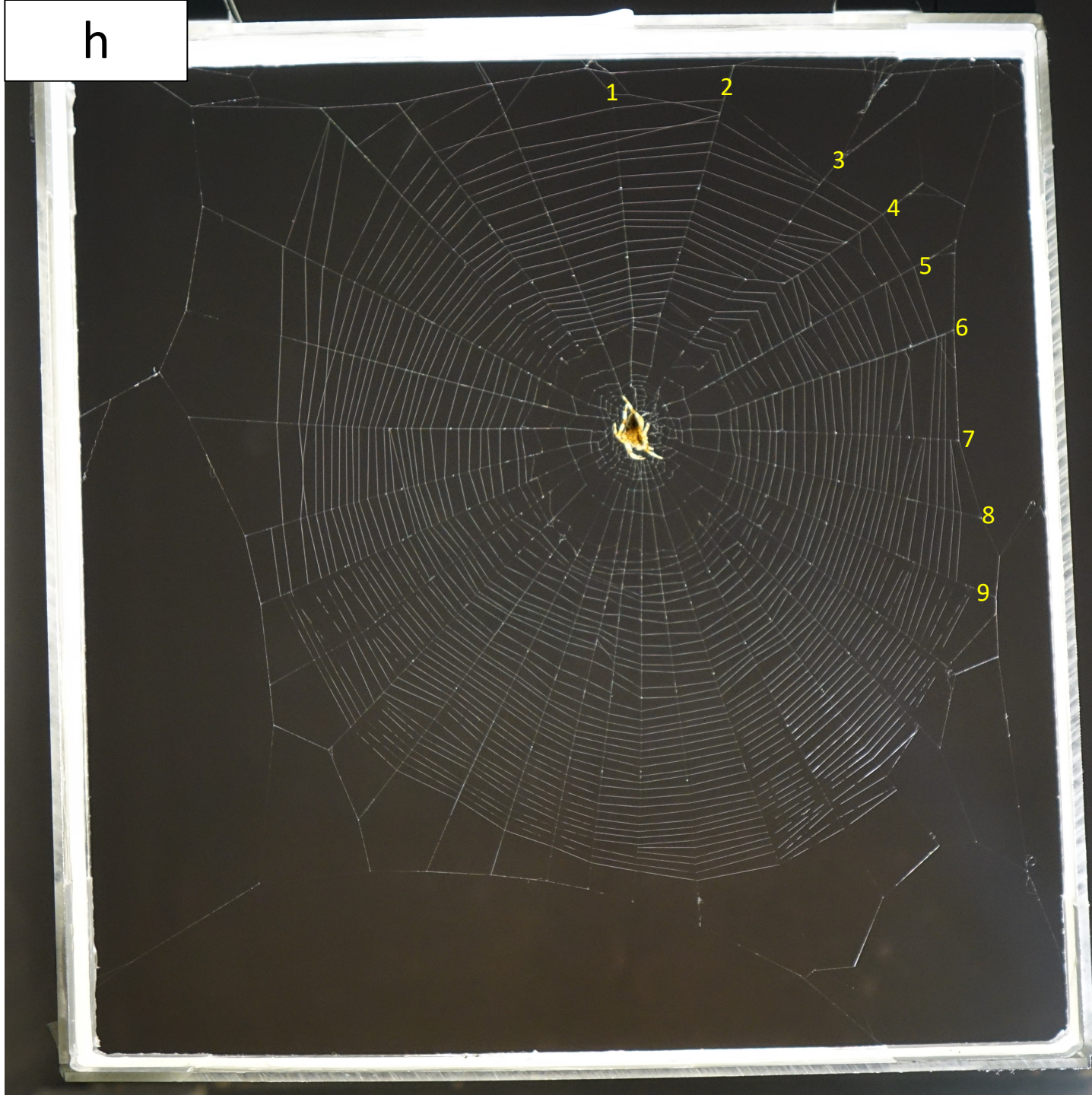

Supplement: Supplementary file 4 — Web-design measurements obtained in ImageJ. a total web area, b hub + capture spiral area, c hub area, d radial length North (yellow), East (green), South (red) West (blue), e capture spiral count North and South (demonstrating North 1–4), f capture spiral width North (yellow) and South (red), g radial count (demonstrating 1–9) (PDF 3367 kb) [file 114_2021_1725_MOESM4_ESM.pdf]

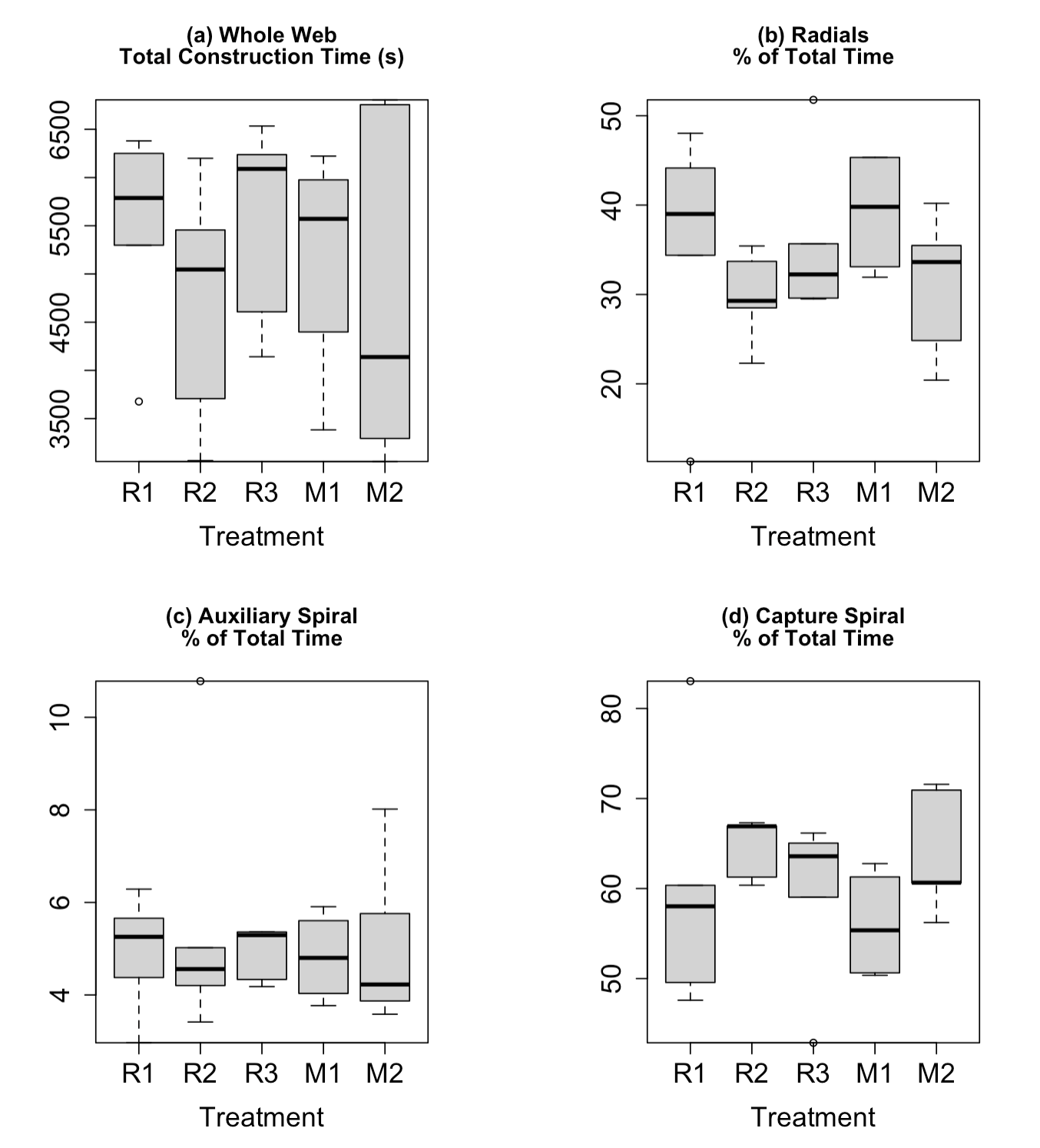

Supplement: Supplementary file 5 — Boxplots demonstrating the lack an experience effect on total web construction time and the percentage of time dedicated to each web component. Treatment = the specific day within the 5-day experimental regime (R1R2M1R3M2) in which the frame is either rigid (R) or moving (M). R-days and M-days are shown sequentially for easy comparison within each treatment group. Plots are based on raw, non-transformed data. The temporal outlier was excluded per the methods on page 11. nrigid = 15, nmoving = 9 (PNG 147 kb) [file 114_2021_1725_MOESM5_ESM.png]

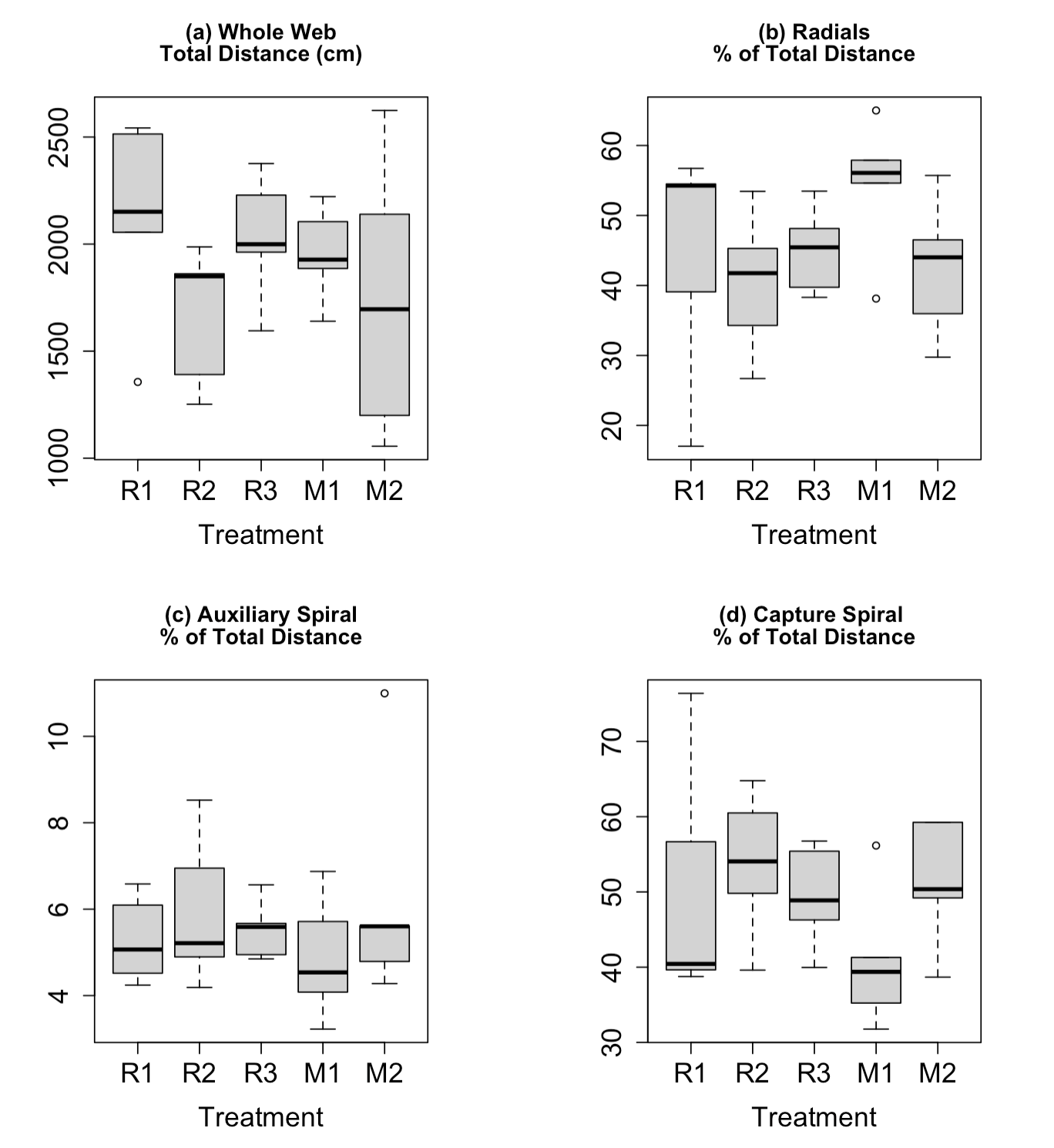

Supplement: Supplementary file 6 — Boxplots demonstrating the lack an experience effect on total distance covered during web construction, and the percentage of total distance covered for each web component. Treatment = the specific day within the 5-day experimental regime (R1R2M1R3M2) in which the frame is either rigid (R) or moving (M). R-days and M-days are shown sequentially for easy comparison within each treatment group. Plots are based on raw, non-transformed data. nrigid = 15, nmoving = 10 (PNG 179 kb) [file 114_2021_1725_MOESM6_ESM.png]

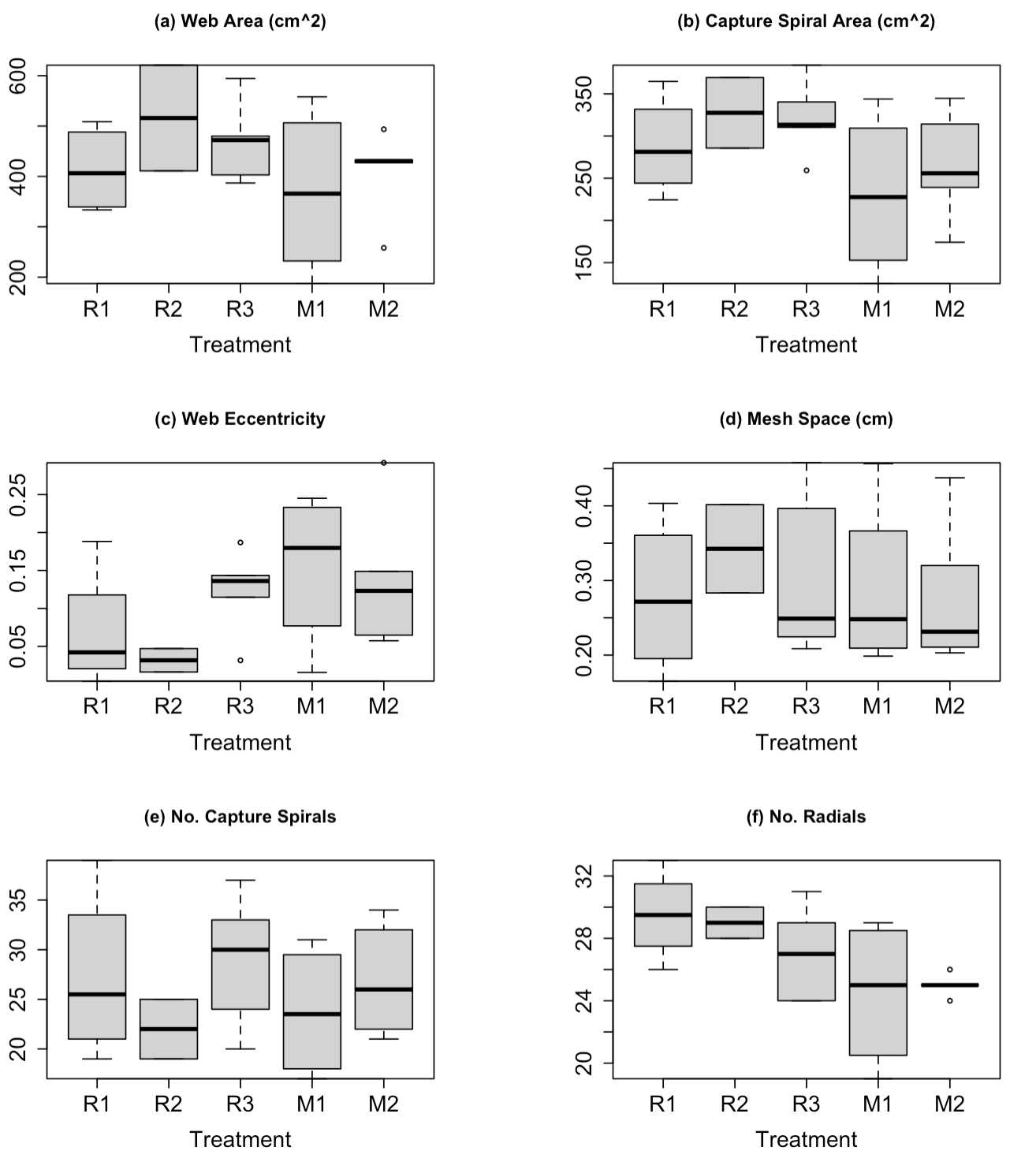

Supplement: Supplementary file 7 — Boxplots demonstrating the lack an experience effect on six web geometry features. Treatment = the specific day within the 5-day experimental regime (R1R2M1R3M2) in which the frame is either rigid (R) or moving (M). R-days and M-days are shown sequentially for easy comparison within each treatment group. Plots are based on raw, non-transformed data. nrigid = 11, nmoving = 9 (PNG 183 kb) [file 114_2021_1725_MOESM7_ESM.png]

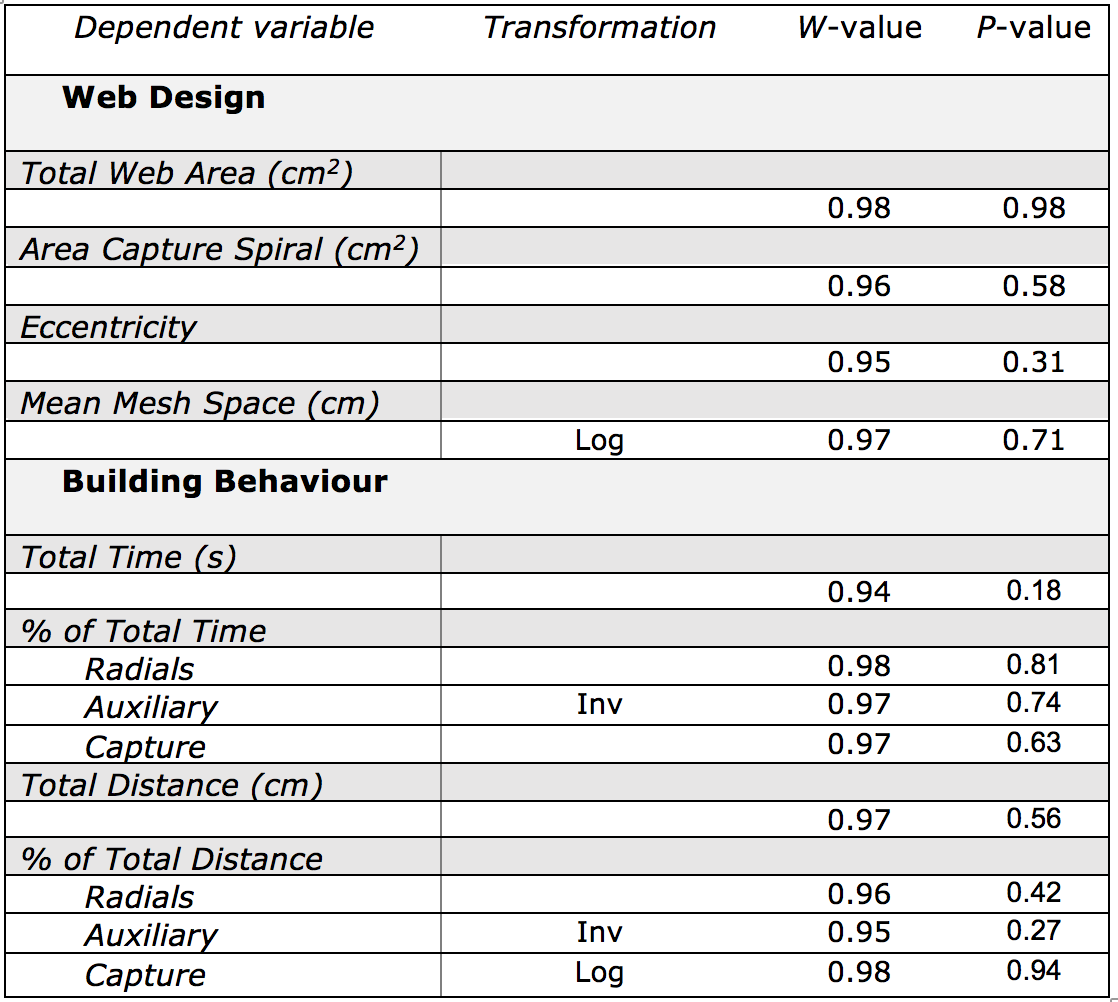

Supplement: Supplementary file 8 — Data transformation employed per variable and Shapiro Wilk test results of LMM residuals. Log log-transformed, Inv inverse transformed (i.e. x-1). Where no transformation is indicated, data were not transformed (PNG 159 kb) [file 114_2021_1725_MOESM8_ESM.png]
